# Effects of an Anticarcinogenic Bowman-Birk Protease Inhibitor on Purified 20S Proteasome and MCF-7 Breast Cancer Cells

**DOI:** 10.1371/journal.pone.0086600

**Published:** 2014-01-27

**Authors:** Larissa da Costa Souza, Ricardo Camargo, Marilene Demasi, Jaime Martins Santana, Cézar Martins de Sá, Sonia Maria de Freitas

**Affiliations:** 1 Laboratory of Biophysics, Department of Cellular Biology, University of Brasília, Brasília, Brazil; 2 Laboratory of Microbiology Department of Cellular Biology, University of Brasília, Brasília, Brazil; 3 Laboratory of Biochemistry and Biophysics, Butantan Institute, São Paulo, Brazil; 4 Laboratory of Pathogen-Host Interface, Department of Cellular Biology, University of Brasília, Brasília, Brazil; Stanford University, United States of America

## Abstract

Proteasome inhibitors have been described as an important target for cancer therapy due to their potential to regulate the ubiquitin-proteasome system in the degradation pathway of cellular proteins. Here, we reported the effects of a Bowman-Birk-type protease inhibitor, the Black-eyed pea Trypsin/Chymotrypsin Inhibitor (BTCI), on proteasome 20S in MCF-7 breast cancer cells and on catalytic activity of the purified 20S proteasome from horse erythrocytes, as well as the structural analysis of the BTCI-20S proteasome complex. *In vitro* experiments and confocal microscopy showed that BTCI readily crosses the membrane of the breast cancer cells and co-localizes with the proteasome in cytoplasm and mainly in nucleus. Indeed, as indicated by dynamic light scattering, BTCI and 20S proteasome form a stable complex at temperatures up to 55°C and at neutral and alkaline pHs. In complexed form, BTCI strongly inhibits the proteolytic chymotrypsin-, trypsin- and caspase-like activities of 20S proteasome, indicated by inhibition constants of 10^−7^ M magnitude order. Besides other mechanisms, this feature can be associated with previously reported cytostatic and cytotoxic effects of BTCI in MCF-7 breast cancer cells by means of apoptosis.

## Introduction

Proteases are involved in many biological processes such as the hydrolysis of intracellular proteins, transcription, cell cycle, cell invasion and apoptosis [1]. The activity of these proteases can be regulated by proteolytic degradation and inhibitors that display variable degrees of affinity with the enzymes [2], [3]. Natural protease inhibitors are classified into about 20 different families [4], [5], among which the Bowman-Birk inhibitors (BBI) and Kunitz have been the most studied [6], [7].

Bowman-Birk inhibitors are found in mono and dicotyledons, especially in leguminous seeds [8]. Diets rich in these legumes have been associated with low incidence of cancer in human populations, in which protease inhibitors are considered to be responsible for this protective action [9]–[11]. In addition, BBIs are the most characterized inhibitors for their role as carcinogenesis suppressors [12]–[16], and they have been studied in a human phase IIa clinical trial [17].

The Black-eyed pea Trypsin/Chymotrypsin Inhibitor (BTCI) is a natural plant protease inhibitor isolated from *Vigna unguiculata* (Cowpea) seeds, and it belongs to the BBI family. Members of this protease inhibitor family are proteins that inactivate the functions of serine proteases by providing a reactive site, present in the canonical loop connecting the β-hairpin motif, which acts competitively as a pseudo or analogue substrate for the cognate enzyme [2],[18],[19]. The remarkable complementarities of these inhibitors, in particular BTCI, determine their high affinity for cognate enzymes. The dissociation constants of 10^−7^–10^−9^ M magnitude order for BBIs and BTCI are compatible with their low dissociation process from the S1 active site of the enzymes [3],[20],[21].

BTCI is a globular protein containing 83 amino acid residues presenting seven disulfide bonds and molecular weight of 9.1 kDa [22]–[24]. It has two different and independent reactive sites for trypsin (Lys26) and chymotrypsin (Phe53) [23]–[26]. Its binary and ternary complexes with these proteases were isolated and physicochemically characterized by analytical ultracentrifugation, viscometry and light scattering, which showed the hydrodynamic parameters and high stability of these complexes at pH 7.0 [25].

The binding constants were calculated by enzymatic assays resulting in values of 10^7^–10^9^ M^−1^ magnitude for chymotrypsin and trypsin, respectively [27],[28]. Additionally, thermodynamic parameters calculated for the formation of trypsin-BTCI and chymotrypsin-BTCI complexes characterized these associations as endothermic, spontaneous and entropy-driven processes [27]–[28]. In spite of the slow process of peptide bond cleavage in the P1 reactive sites of BTCI and the characteristic reversibility of the inhibition process, the presence of one disulfide bond flanking each loop containing the P1 residues prevents the displacement of the product from the S1 enzyme pocket [24].

The biochemical, biophysical and biotechnological properties of BTCI have been extensively characterized [14],[23],[24],[27]–[35]. BTCI is a thermally stable protein that retains 96% of its inhibitory activity after heating at 95°C for 60 min, as well as when it is exposed from pH 3 to 10 [30]. BTCI presented *in vitro* and *in vivo* effects on development of the boll weevil (*Anthonomus grandis*), a cotton pest. It caused larval growth delay and reduced the insect population, showing its potential for expression in transgenic plants engineered for pest resistance [31]. Dose-response experiments showed that BTCI was capable of inducing changes in renal functions by enhancing guanylin-induced natriuresis, a strong but transient diuretic response. Additionally, BTCI promoted increases in urine flow, in fractional excretion of Na^+^ and K^+^, in perfusion pressure, in glomerular filtration rate and osmolar clearance [32]. It was the first description of renal effects induced by a protease inhibitor belonging to the Bowman-Birk family. Both effects are due to the ability of BTCI to inhibit chymotrypsin-like protease, thus playing a fundamental role in the renal metabolism of guanylin in a natriuretic process [32] and trypsin- and chymotrypsin- like protease from the midguts of larvae and adult insects from the cotton boll weevil *Antonomus grandis* [31]. Furthermore, our previous results indicated that BTCI induced significant cytostatic and cytotoxic effects on MCF-7 breast cancer cells associated with severe cell morphological alteration, lysosome membrane permeabilization and apoptosis [14]. Although BTCI induced a significant reduction in MCF-7 cancer cell viability and proliferation (arrest in S and G2/M phase), it did not affect the viability of normal MCF-10A breast cells. BTCI induced severe morphological changes in MCF-7 cancer cells, such as plasma membrane fragmentation, cytoplasm disorganization, and presence of double-membrane vesicles, mitochondrial swelling, and an increase in the size of lysosomes. In addition, meaningful DNA fragmentation, annexin-V+ cell number increase, mitochondrial membrane potential reduction, and cytoplasm acidification were also detected [14].

In another study, it was shown that topical applications of BTCI on the skin of mice significantly reduced the incidence and volume of pre-malignant lesions during chemical induction of non-melanoma cancer in this tissue [35]. Reduction was also observed both in the number of histopathological features as well as in production of inflammatory mediators involved in tumor progression. Regarding the preventive effects, BTCI treatments were able to retard the progression of non-melanoma skin cancer, probably inducing anti-inflammatory effects [14],[35]. Non-melanoma skin and breast cancer are of high incidence, and breast cancer is the most frequent malignancy among women worldwide [36]; both are leading causes of cancer-related mortality.

The high stability of BTCI in terms of maintaining its inhibitory activity at temperatures as high as 95°C in a wide range of pHs (3–11), besides its lack of effect on normal breast cells MCF-10A, are of fundamental importance in evaluating the reduction of side effects using, in the future, BTCI in breast cancer treatment. Therefore, the aforementioned results are relevant in focusing on BTCI as a potential anticarcinogenic drug, so as to design new strategies for current breast cancer treatments, as well as skin cancer prevention.

As is known, cancer is related to dysfunction in the cell cycle in which the ubiquitin-proteasome system plays a fundamental role in the proteolysis of intracellular proteins [37],[38]. The 20S proteasome is a catalytic complex mediating caspase-like, trypsin-like and chymotrypsin-like activities in the β1, β2, and β5 catalytic subunits, respectively [39], important in ubiquitinated protein breakdown. This process occurs after recognition of ubiquitinated proteins by 26S proteasome [40]–[42], which is formed by 20S proteasome binding to one or two 19S regulatory caps [43].

Cancer cells are generally associated with increased constitutive proteasome activity compared to non-malignant cells, due to cell proliferation, increased oxidative stress, and elevated cytokine levels, which can also induce the expression of immunoproteasomes [44]. The constitutive 20S proteasome is present in all eukaryotic cells, whereas the immunoproteasome is mainly expressed in immunocompetent tissues. The β1, β2, and β5 catalytic subunits present in the constitutive proteasome are replaced by β1i (low-molecular-weight protein-2, LMP2), β2i (multicatalytic endopeptidase complex–like-1, MECL1) and β5i (LMP7) in the immunoproteasome [45],[46]. Despite both proteasome diversities, the expression and function of the immunoproteasome in different cancer types seem to be discrepant in the literature and still remain under investigation [47]. Regarding MCF-7 breast cancer cells, few descriptions of the expression and activity of the immunoproteasome subunits have been reported [48]–[50]. As shown, in MCF-7 cells both β5i/LMP7 and β1i/LMP2 subunits presented minimum expression levels when compared to other cancer cells, even with MCF-10A cells, indicating that immunoproteasomes are almost absent in MCF-7 breast tumor cells [48].

Proteasome inhibition results in cellular homeostasis disruption [51] and in the induction of apoptosis [52],[53]. In this context, proteasome inhibitors have been characterized as important compounds for cancer therapy [54]. Among them, Bowman-Birk inhibitors (BBI) appear as a very promising compound of anticarcinogenic drugs. As reported in 2005 [55], BBI from soybean inhibits the chymotrypsin activity of the proteasome in breast cancer cells, but the molecular mechanism involved in this process is not fully understood. BBI in MCF-7 cells decreases proteasome function and results in up-regulation of MAP kinase phosphatase-1, which suppresses ERK1/2 activity. These results led to BBI being indicated as a novel mechanism that contributes to prevention of cancer, as well as a potential chemopreventive agent [55].

Here, we report the effects of BTCI on the 20S proteasome of MCF-7 breast cancer cells and on the catalytic activity of the 20S proteasome purified from horse erythrocytes, as well as the molecular analysis of the BTCI-proteasome complex. Our findings also identified BTCI as a proteasome inhibitor, which could be related to ubiquitin-proteasome pathway inhibition and its previously reported anticarcinogenic effects [14].

## Materials and Methods

### Protein Purification

BTCI was purified as previously described [29] and its purity was confirmed by mass spectrometry analysis [30]. The 20S proteasome used in enzymatic assays, BTCI-proteasome association and structural stability studies was purified from horse erythrocytes. The purification was performed by sequential chromatography in DEAE-Sepharose, sucrose density gradient ultracentrifugation and chromatography on mono-Q column using FPLC facility (Pharmacia) [56]. This proteasome source was chosen considering its well established purification procedure resulting in a high amount of protein and also the conserved structural features of the proteasome in all organisms. For all experiments, BTCI and 20S proteasome were sterilized by filtration with a 0.22 µm membrane (Millipore, USA).

### BTCI and 20S Proteasome Association Evaluated by Dynamic Light Scattering (DLS)

The interaction of BTCI with 20S proteasome was analyzed by DLS assays carried out through a laser wavelength of 800 nm, using a DynaPro - DLS model (Wyatt Technology Corporation, Santa Barbara, CA, USA) molecular-sizing instrument equipped with a Peltier system for temperature control. Solutions of proteins were centrifuged at 15,000 *g* for 20 min at 4°C, and the supernatant filtered through a 0.22 µm filter (Millipore) and added to the cuvette. The hydrodynamic parameters were measured at different pHs in 20.0 mM buffers (KCl pH 2.0; glycine HCl pH 3.0; sodium acetate pH 4.0–6.0; Tris-HCl, pH 7.0–9.0; glycine NaOH, pH 10.0–12.0), temperature range of 25–60°C and protein concentration of 21.0 nM for 20S proteasome and 15.0–90.0 µM for BTCI.

The intensity of scattered light from each sample was normalized considering the buffer scattering contribution. Polydispersity (*Pd*), hydrodynamic diameter (*D_H_*) and molecular weight were determined from the intensity correlation function using the cumulant method [57],[58] and the Dynamics V.6 software. The experiments were performed with an average of 100 acquisitions.

### Cell Culture

Human MCF-7 breast cancer cells, from American Type Cell Collection, were grown in Dulbecco’s modified Eagle’s medium (DMEM) (HyClone, Logan, UT USA) containing 10% fetal bovine serum (FBS – Invitrogen, USA), 30.0 µg/mL streptomycin (Invitrogen, USA) and 100.0 µg/mL ampicillin (Invitrogen, USA). Cells were maintained in a humidified atmosphere of 5% CO_2_ at 37°C.

### Antibody Anti-BTCI Preparation

The antibody against BTCI was obtained from immunized Swiss mice lineage with 3 applications of purified BTCI (0.1 µg/µL) in phosphate-buffered saline (PBS) (10 mM phosphate, 137 mM NaCl, 2.7 mM KCl, pH 7.4) and complete adjuvant, then with incomplete adjuvant and without adjuvant, sequentially. The blood was collected from the carotid vein after anesthetization with ketamine (75 µl) and xylazine (120 µl) in PBS (805 µl) and then clotted and centrifuged at 10,000 g for 7 minutes in order to obtain the serum. A polyacrylamide gel with 400 µg of BTCI was transferred to a nitrocellulose membrane and incubated for 2 hours with the serum anti-BTCI. The membrane was washed with PBS and the antibody was eluted with 0.2 mM glycine-HCl buffer at pH 2. The solution containing the antibody was adjusted to pH 7.0 and concentrated in amicon ultra-0.5 mL centrifugal filter (Merck Millipore). The functionality of the antibody was confirmed by Western blotting. This study was approved by the Ethics Committee (Proc. No 47028/2007) of the Institute of Biology, University of Brasilia.

### Immunofluorescence Analysis

MCF-7 cells were seeded on 12-well plates at a density of 5×10^4^ cells in culture medium and incubated with 200 µM BTCI for 2, 6, 12 and 24 h at 37°C. The negative control was done with cells incubated in the absence of BTCI. Cells were harvested, washed three times with phosphate-buffered saline (PBS) and fixed with 3.7% paraformaldehyde. Cells were permeabilized with 0.2% Triton-X in PBS, blocked with PBS - 5% of skimmed milk (w/v) and incubated with primary antibodies: anti-BTCI at 1∶100 dilution (serum obtained from immunized mice with purified BTCI), and rabbit anti-20S proteasome core (Affiniti – Biomol, USA) in PBS - 1% skimmed milk (w/v), at room temperature for 2 h. Cells were washed with PBST (PBS, 0.2% Tween 20, v/v - Pharmacia Biotech, Buckinghamshire, UK). Goat anti rabbit Alexa Fluor 633-conjugated and goat anti mouse Alexa Fluor 488-conjugated secondary antibodies (Invitrogen, USA), at 1∶400 dilutions, were incubated in PBS - 1% skimmed milk (w/v), for 2 h, in dark room, at room temperature. A blue nuclear counterstain 4′,6-diamidino-2-phenylindole (DAPI) was used to visualize nuclear DNA in fixed cells. Cells were washed with PBST and MilliQ water, and then analyzed in Immunofluorescence Confocal Microscopy (Leica Microsystems, TCS SP5 German). The fluorescence intensity of the images was quantified by ImageJ program (Wayne Rasband; Research Services Branch, National Institute of Mental Health, Bethesda, Maryland, USA). All values were expressed as mean ± SD of three independent experiments. The differences in the fluorescence intensity of each group were analyzed by *t*-test. Asterisk indicates that the values are significantly different from the group analyzed (*, p<0.001).

### Enzymatic Assays

Purified 20S proteasome at concentration of 2.0 µg/mL was incubated with BTCI (2.0 to 30.0 µg/mL) and 62.5 µg/mL of the following fluorogenic substrates in 20.0 mM Tris-HCl pH 7.5, 1.0 mM EDTA, 1.0 mM NaN_3_, 1.0 mM DTT: *t-butyloxycarbonyl- L-leucyl-L-arginyl-L-arginine-4-methylcoumaryl-7-amide* (Boc-leu-arg-arg-AMC) for trypsin-like; *benzyloxycarbonyl-L-leucyl-L-leucyl-L-glutamyl-naphthylamide* (Z-leu-leu-glu-βna) for caspase-like and *N-succinyl-Leu-Leu-Val-Tyr-AMC* (Suc-leu-leu-val-tyr) for chymotrypsin-like activities. The control of proteasome inhibition assays was done using the proteasome inhibitor named MG132 (carbobenzoxyl-leucyl-leucyl-leucinaI-H, also called Z-LLL-CHO) at a similar concentration to that at which BTCI showed ∼80–95% of proteases inhibition. The assays were carried out in triplicate, at room temperature for 60 min. The hydrolysis of fluorogenic substrates was monitored at 480 nm (wavelength of emission, λ_em_), after excitation at 380 nm (λ_exc_) for chymotrypsin-like and trypsin-like, and λ_exc_ of 410 and λ_em_ of 335 nm for caspase-like. Inhibition curves were obtained by plotting the relative activities of the proteases *versus* BTCI concentration. Inhibition constant of the enzyme-inhibitor complex, *K_i_*, was calculated from fitted inhibition curve considering the Morrison equation using the GRAFIT program version 3 (Erithacus Software Ltd., UK). All inhibition constants were calculated until the chi-square (χ^2^) reached a value less than 0.012, compatible with the best fit curves representing the experimental data. The control for this experiment was done using the proteasome inhibitor named MG132 at a similar concentration to that used when BTCI showed ∼80–95% of protease inhibition.

## Results and Discussion

### BTCI and 20S Proteasome Association Evaluated by Dynamic Light Scattering

The hydrodynamic parameters of the 20S proteasome in complex with BTCI, as well as the self-association tendency of BTCI and the proteasome, in a temperature and pH dependent manner, were investigated by using dynamic light scattering. This technique has been commonly used mainly to analyze protein crystallization tendency [59]. Furthermore, in the last decade, DLS has also been a useful tool in a number of structural studies involving proteins and nanoparticles, such as association of proteins with surfactants [60], protein unfolding process [61], protein stability [62],[63], dimension of nanoparticles [64]–[66], protein-protein interaction and protein self-association process [67]–[70], in addition to others.

**Figure 1:**
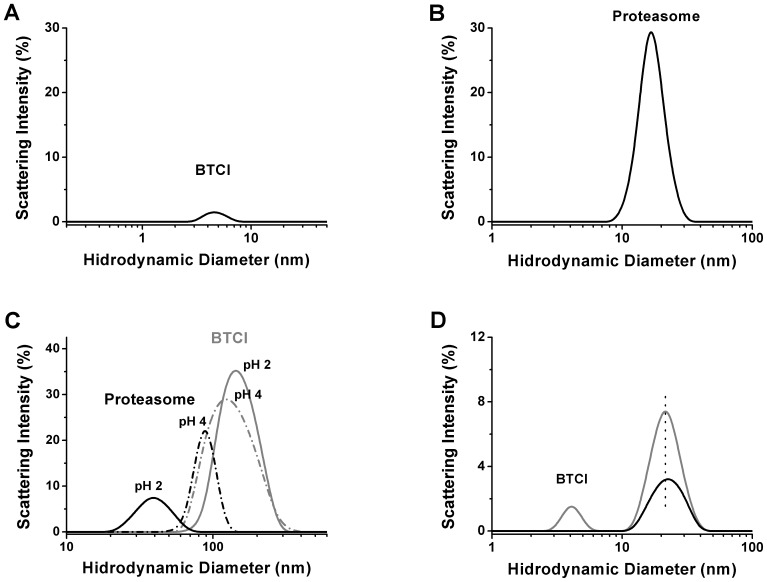
Dynamic Light Scattering size distribution of BTCI and the proteasome. Hydrodynamic diameter of BTCI as a trimer (15 µM; 25 kDa) and the 20S proteasome (21.4 nM; 505,2 kDa) as a monomer at neutral pH (A and B) and as aggregate in acidic condition (C) determined by Dynamic Light Scattering. The BTCI and BTCI-proteasome complex (in gray) were shown in panel (D). The conformational change of the complex (in black) was observed as indicated by differences in scattering intensity, polydispersity and hydrodynamic diameter (from 21.8 nm to 22.7 nm).

In the present work, the parameters obtained from DLS measurements indicate that the 20S proteasome appears as a monomer at 21.4 nM and pH 7.5 with hydrodynamic diameter of 15 nm (Fig. 1a), in agreement with previously reported data [71],[72]. BTCI forms a trimer at 15 µM (Fig. 1b) and pH 7.5, with hydrodynamic diameter of 4.5 nm, which is consistent with the tendency of this inhibitor to form oligomers [33],[73]. In contrast, at pH 2.0 and 4.0 (Fig. 1c) BTCI and 20S proteasome assembled into oligomeric or aggregate structures. BTCI interacts with the 20S proteasome in the first 15 min and, after that, induces low conformational changes in the 20S proteasome, as indicated by the increase in the hydrodynamic diameter of the complex from 21.8 nm to 22.7 nm (Fig. 1d) and concomitant disappearance of scattering peak corresponding to BTCI.

**Figure 2:**
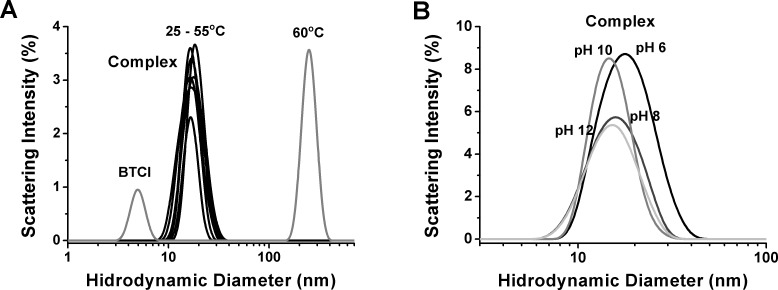
Dynamic Light Scattering size distribution of BTCI-proteasome complex. A) Hydrodynamic diameter of the BTCI-proteasome complex as a function of temperature and B) pH. The complex appears as a stable protein until 55°C but dissociates and forms BTCI as a trimer and the proteasome as an aggregate up to 60°C. The conformational changes in the complex were observed as a function of pH, as indicated by the differences in hydrodynamic diameter.

The proteasome-BTCI complex presented tertiary conformational changes as indicated by differences in hydrodynamic diameters under these conditions (Fig. 2a). Indeed, the complex was characterized as thermally stable at pH 7.5 and up to 55°C, but dissociated and formed an aggregate above 60°C (Fig 2b). These structural changes were attributed mainly to the proteasome, since BTCI was previously reported as a highly thermostable protein within a wide pH range (3.0–10.0), preserving its tridimensional structure and inhibitory activity up to 95°C after incubation for 60 min [30].

The structure of the 20S proteasome is conserved through fungi to humans [74]. Its physicochemical features, such as sedimentation and diffusion coefficients, secondary structure content and amino acid composition, are similar among eukaryotic organisms [75]. The 20S proteasome was here characterized as a stable protein complex from pH 6.0 to 12.0, with aggregation tendency at acid pH, and structural stability up to 60°C, where dissociation of proteasome ring structures probably occurs. Our finding corroborates previous data reported for the 20S proteasome from ostrich skeletal muscle, which presented pH and temperature optima ranged between 8.0 and 11.0, and 40 to 70°C, respectively, and stabilities for enzymatic activities between pH 5.0 and 12.0 up to 60°C [76].

### Immunocolocalization of BTCI and Proteasome in MCF-7 Breast Cancer Cells

**Figure 3:**
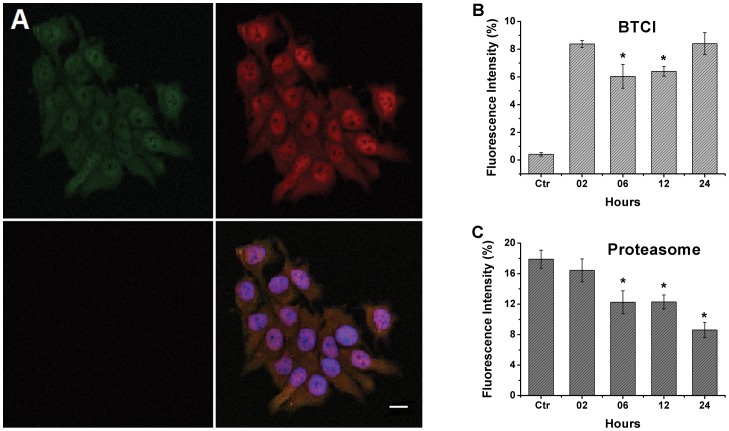
Immunofluorescence of the proteasome and BTCI in the MCF-7 cells by Confocal Microscopy. A) BTCI (green), proteasome (red), and BTCI proteasome colocalization (yellow) with DAPI DNA marked in blue. The anti BTCI specificity was assessed in MCF-7 cells treated with 200 µM BTCI for 2, 6, 12 and 24 hours, whereas the control was done without BTCI (black). Fluorescence intensity of BTCI (B) and the proteasome (C) was calculated by ImageJ program as a function of the incubation time of BTCI in MCF-7 cells. Ctr: control; T-test: *p<0.001, compared to 2 hours of BTCI incubation. Bar = 20 µm.

The immunocolocalization of BTCI and the proteasome in MCF-7 breast cancer cells was investigated by confocal microscopy. Cells were treated with 200 µM BTCI from 2 to 24 h. The negative control was performed in the absence of the inhibitor. Figure 3a shows that BTCI (green) and the proteasome (red) are distributed all over the cells after 2 h incubation. The two molecules co-localize in the cytoplasm and nucleus of cells, as indicated by the yellow color, corresponding to the overlapping Alexa Fluor 488 (green) and Alexa Fluor 633 (red) antibody conjugated fluorophores, respectively. These results indicate that MCF-7 cellular membranes are very permeable to BTCI, and that once BTCI is inside cells, it co-localizes in similar regions, such as the proteasome.

The anti-20S proteasome used in the present study was a general antibody that is able to detect multiple constitutive (β1, β2, β5) and immunoproteasome (β1i, β2i, β5i) subunits. The co-localization observed in the immunofluorescence assay (Figure 3a) using the anti-20S core antibody probably occurs between BTCI and the constitutive proteasome. It is supported by the reportedly very low expression of immunoprotesomes in MCF-7 cells [48]–[50]; hence, their representation in this assay is negligible. Therefore, the detection of i20S or BTCI targeting the i20S subunits in MCF-7 cells could be difficult to verify.

Fluorescence intensity quantification (Fig. 3b–c) was done by recording the average of the fluorescence intensity of each tagged protein using the *ImageJ* program (Wayne Rasband; Research Services Branch, National Institute of Mental Health, Bethesda, Maryland, USA). The fluorescence intensity of BTCI (Fig. 3b) decreased through the first 12 h (p<0.001) and increased at 24 h (p<0.001), when compared with the first 2 h of incubation. Moreover, the fluorescence intensity of the proteasome (Fig. 3c) decreased significantly in the presence of the inhibitor (p<0.001).

This result suggests that BTCI enters through the MCF-7 cell (high initial fluorescence intensity with 2 h incubation) compatible with continuous flux of the inhibitor through the cell, coincident with cellular membrane disruption and other morphological alterations in the cell, as previously reported by Joanitti *et al*. (2010) [14]. In contrast, the fluorescence intensity corresponding to the proteasome (Fig. 3c) decreased until 12 h, which may have occurred as a consequence of the steric hindrance of antibody binding due to conformational changes in the proteasome caused by BTCI.

These immunofluorescent assays showed that this inhibitor is taken up by the MCF-7 cells in a time-dependent manner and is present in the cells for 24 h. In addition, according to our previous results, the ultra-structural analysis of MCF-7 cell morphology indicated a pronounced effect of BTCI on plasma membrane fragmentation, cytoplasm disorganization, presence of double-membrane vesicles, and lysosome size increase [14]. The recognition and internalization processes of BTCI by MCF-7 are unknown. However, according to those results, this process can be activated after structural and/or functional alterations of plasma membrane integrity occurred with exposure of phosphatidyl serine outside the inner membrane.

### BTCI Inhibits the 20S Proteasome Catalytic Activities

**Figure 4:**
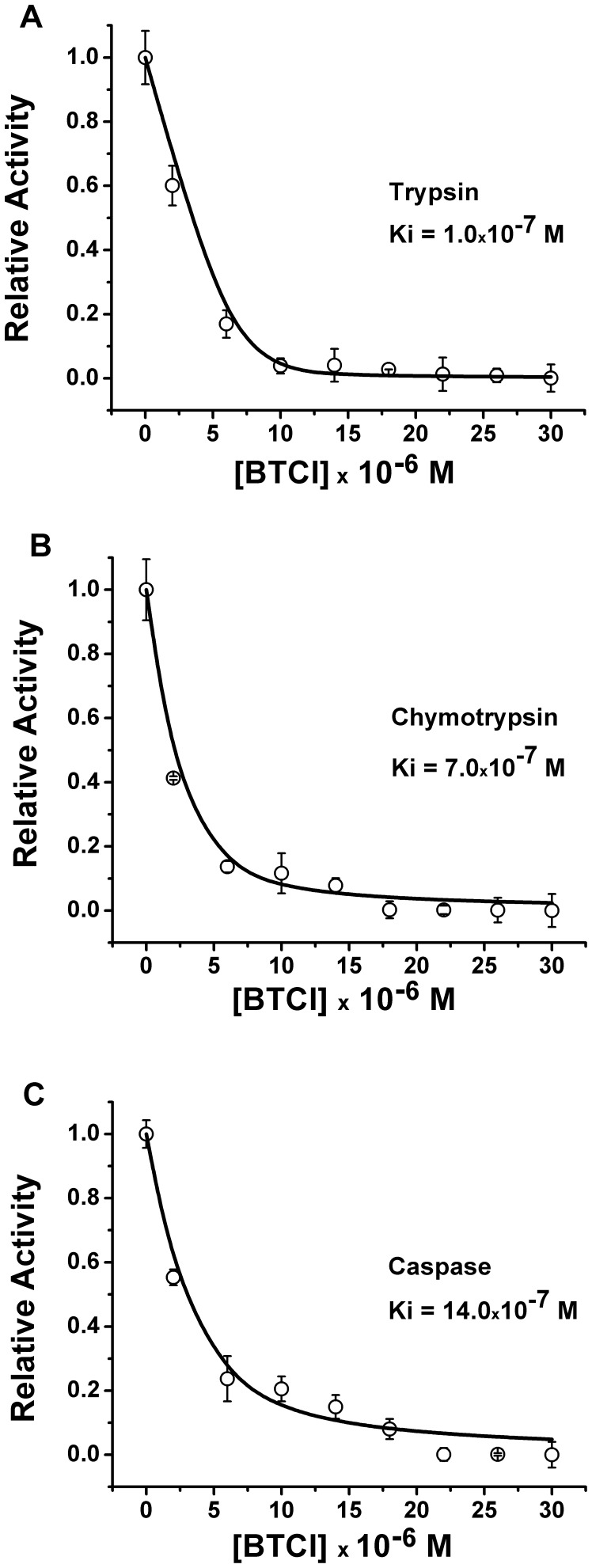
Inhibitory activity of BTCI toward the 20S proteasome. A) Relative activity of proteasome trypsin-like; B) Relative activity of proteasome chymotrypsin-like; C) Relative activity of proteasome caspase-like. The dissociation or inhibition constants, calculated from the fitted curve by GraFit program (Erithacus software Ltd.), were 1.0×10^−7^ M, 7.0×10^−7^ M, and 14.0×10^−7^ M, respectively.

**Figure 5:**
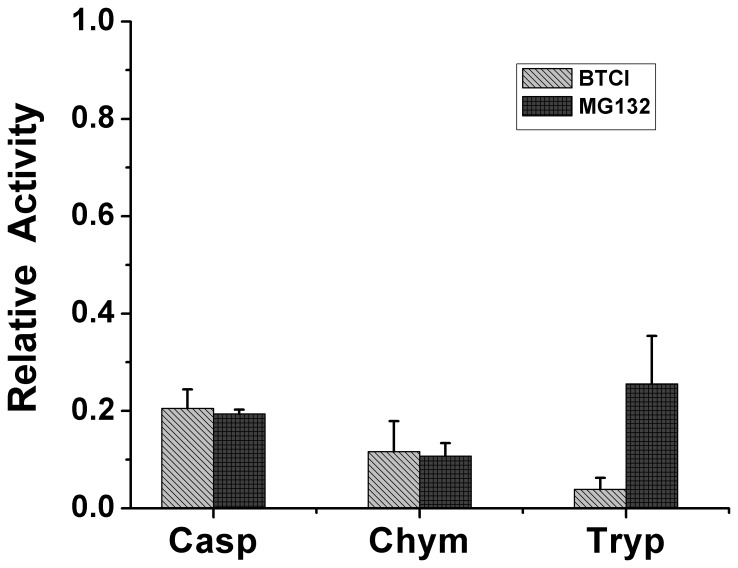
Inhibitory activity of BTCI and MG132 (positive control), toward 20S proteasome caspase-like (Casp), chymotrypsin-like (Chym) and trypsin-like (Tryp). The concentration of both inhibitors is 10 µM. As shown, BTCI is a more potent inhibitor for trypsin than MG132 and presented similar inhibition to MG132 against caspase-like and chymotrypsin-like.

Three protease activity sites are present in the β subunits of the 20S proteasome, including the caspase-like (β1), trypsin-like (β2) and chymotrypsin-like (β5) sites [77]–[79]. In the present work BTCI was characterized as a novel and potent Bowman-Birk inhibitor of the 20S proteasome through specific inhibition of those three protease activities. BTCI presented high affinity to the 20S proteasome, as indicated by inhibition or dissociation constants (K_I_ or K_d_) values of 1.0×10^−7^ M, 7.0×10^−7^ M and 14.0×10^−7^ M for trypsin-like (Fig. 4a), chymotrypsin-like (Fig. 4b) and caspase-like sites (Fig. 4c), respectively. The calculated K_I_ magnitude order of 10^−7^ to 10^−8^ M is similar to the previous estimate for most BBI inhibitors [3],[25],[80] and also BTCI [27],[28]. In addition, BTCI was able to inhibit all proteases in a similar way to the known proteasome inhibitor MG132 (carbobenzoxyl-leucyl-leucyl-leucinaI-H), here used as a control of proteasome inhibition assays (Fig. 5). It can be observed that BTCI was a more potent inhibitor for trypsin than MG132 and presented a similar inhibition to MG132 against caspase- and chymotrypsin-like activities.

MG132 was among the first developed proteasome inhibitors and the most widely used in research. It is a peptide aldehyde-based reversible proteasome inhibitor, which inhibits the proteasome primarily on the chymotrypsin-like site, but also inhibits trypsin- and caspase-like sites. Although it is a potent proteasome inhibitor, MG132 is rapidly oxidized into inactive carbonic acid *in vivo* and, for this reason, its therapeutic use is usually prevented [81]–[83].

BTCI was the first member of the Bowman-Birk family to be characterized as a potent inhibitor of all three trypsin-like, chymotrypsin-like and caspase-like proteasomal activities. As previously reported, BBI, a Bowman-Birk Inhibitor isolated from soybean, inhibits only chymotrypsin-like activity (inhibition of 70%) of the 26S proteasome *in vitro* at 40 µM [55]. In contrast, BTCI inhibited almost 100% of the three enzymatic activities of the 20S proteasome at 20 µM. This indicates that although BTCI and BBI present similar structures [24], low differences in primary and tertiary structure of BTCI, compared to BBI, are essential for its significant inhibition of three proteasome proteases.

Surprisingly, the way that BTCI inhibits the caspase-like proteasomal activity has never been reported before in the case of any other Bowman-Birk type inhibitor. This ambiguous feature is sustained by the structural changes in the 20S proteasome which occurred when in complex with BTCI (Fig. 1c). The BTCI-20S proteasome complex probably favors the steric hindrance effects, where the caspase-like catalytic site may be buried within a large protein surface, when BTCI binds specifically to chymotrypsin-like or trypsin-like catalytic sites. These conformational changes reflect the differences in proteasome enzymatic activities in the presence of their specific substrates and BTCI. This is in accordance with previous work reporting the conformational changes in the proteasome when a particular active site is activated or inhibited [84].

Studies of proteasome inhibitors and yeast mutants have shown that chymotrypsin-like activity is a rate-limiting step responsible for the ubiquitinated protein breakdown [85]–[89]. In mammalian cells, the caspase-like and trypsin-like sites also play a significant role in protein degradation, but the relative importance of the three active sites depends on the protein being degraded. Indeed, previous studies show that simultaneous inhibition of the chymotrypsin-like and the caspase- or trypsin-like sites were needed to decrease protein degradation by the proteasome [90]. As discussed, the inactivation of one site is not sufficient to markedly block protein degradation by the proteasome. As previously reported, BTCI can inhibit the activity of chymotrypsin and trypsin enzymes independently and simultaneously through its two specific reactive sites (P1), Lys26 and Phe53, acting as a substrate analogous for cognate serine proteases by forming stable ternary or binary complexes with those enzymes [23]–[25],[27],[28],[34]. Similarly, BTCI can inhibit the activity of all three enzymes of the β1, β2, and β5 subunits of the 20S proteasome.

As is known, BBIs from legumes are potent inhibitors of both trypsin and chymotrypsin, with *K*i values within the nanomolar units [3],[21]. These inhibitors, such as BTCI, are well- characterized as “double-headed” protease inhibitors, presenting two conserved reactive sites located in two opposite and independent domains. The structure of BTCI folds into two similar domains with three β-strands forming an antiparallel β-sheet stabilized by disulfide bonds. Each domain contains one reactive site in position P1, Lys26 and Phe53, located in a loop connecting two strands, capable of binding to the active site of the trypsin and chymotrypsin, respectively. The distance between the Cα atoms of the two reactive sites (32.5 A°) is very similar to that of the BBIs, which allows the inhibition of two protease molecules simultaneously and independently [24],[25]. According to our results, the inhibition of the proteasome by BTCI probably occurs in an independent manner for all three proteases, since their active sites are positioned in the N-terminal region of independent β1, β2 and β5 subunits of the 20S proteasome. In this case, independent monomers of BTCI are oriented towards each specific active site of the different β subunits composing the 20S proteasome. Additionally, it is known that the three β1, β2 and β5 subunits work independently [91].

The magnitude of dissociation constants was similar to those obtained for free BBIs with cognate enzymes. These results indicate that reactive P1 sites of the BTCI bind to the specific S1 pocket of the catalytic region of the subunits β2 (trypsin-like), β5 (chymotrypsin-like) and block the active site of β1 (caspase-like) of the 20S proteasome [92]. This property strongly indicates that this protein can act as a potent inhibitor of the proteasome, one of the most important macromolecular complexes involved in intracellular protein breakdown. These results can partially corroborate recently reported cytostatic and cytotoxic effects of BTCI on MCF-7 breast cancer cells [14]. In this case, the induction of apoptosis cell death by BTCI is associated with severe morphological alterations in the cell and lysosome membrane permeabilization. These alterations are related to degradation of the cytoskeleton proteins, structural protein-phospholipid membrane disruption, chromatin condensation and DNA fragmentation, where cytoplasmatic caspase proteins play vital roles. These proteins belong to a group of enzymes known as cysteine proteases and exist within the cell, differently from the caspase*-*like protein of the proteasome, as inactive pro-forms or zymogens. These zymogens can be cleaved to form active enzymes following the induction of apoptosis [93].

Currently, there are numerous reports of proteasome inhibitors which act only on one or two protease-like sites of the proteasome, but few proteasome inhibitors acting simultaneously on three proteases-like sites [94]. One of the most investigated proteasome inhibitors is bortezomib, the first proteasome inhibitor approved for clinical use by the FDA in 2004, which is a more potent inhibitor for β5 than β1 subunit. Bortezomib is a reversible inhibitor of the proteasome in spite of its very slow dissociation rate [82],[83]. This inhibitor has several molecular effects, such as cell cycle arrest in G2/S phase [95], stabilization of cell cycle regulatory proteins, inhibition of NF-κB activation, induction of apoptosis, among others [96],[97], mainly involving the inhibition of subunit β5 (chymotrypsin-like activity) of the proteasome [98].

Like bortezomib, BTCI is a reversible inhibitor that inhibits the proteases of the β1 and β5 subunits. Moreover, like MG132 (Fig. 5), BTCI inhibits all three chymotrypsin-, trypsin- and caspase-like activities of the 20S proteasome. Although bortezomib and MG132 are considered to be potent proteasome inhibitors, exploratory investigation of others is needed. This is principally due to the occurrence of bortezomib resistance after clinical use in multiple myeloma and other diseases, such as malignant lymphoma [99], and the fact that the therapeutic use of MG132 is usually prevented. In these contexts, therefore, the fact that BTCI inhibits all three chymotrypsin-, trypsin- and caspase-like activities may be an advantage in its application in cancer therapy, compared to other reported proteasome inhibitors. These inhibitory properties of BTCI are probably associated with its previously reported cytostatic and cytotoxic effects on breast cancer cells [14], as discussed above. Furthermore, as is known, the inhibited ubiquitin-proteasome pathway can induce cell death. This is one of the molecular pathways of cell death that need to be investigated in order to verify the involvement of BTCI.

The mechanism by which BTCI induces apoptosis of the MCF-7 cells is not yet known. However, as indicated here, BTCI activity against the 20S proteasome may be related to apoptosis by inhibiting protein breakdown processes. This mechanism, along with others promoting cytostatic and cytotoxic effects, may be responsible for the anticarcinogenic effect of BTCI on MCF-7 cells by means of apoptosis.

Finally, this work is the first study analyzing the molecular association of BTCI with the 20S proteasome in breast cancer pathways. BTCI forms a stable complex with the 20S proteasome, independently inhibiting its three catalytic activities. Our data also indicate that due to its inhibitory action on the proteasome, BTCI may be associated with the anticarcinogenic effects previously reported in MCF-7 cancer cells [14]. Therefore, BTCI can become an even more promising anticancer agent in the treatment and prevention of breast cancer, but other studies should be undertaken to elucidate the mechanism of BTCI action in breast cancer cells.

## References

[1] Kato GJ (1999) Human genetic diseases of proteolysis. Hum Mutat 13: 87–98. 10094566 10.1002/(SICI)1098-1004(1999)13:2<87::AID-HUMU1>3.0.CO;2-K

[2] Laskowski M, Kato I (1980) Protein inhibitors of proteinases. Annu Rev Biochem 49: 593–626. 6996568 10.1146/annurev.bi.49.070180.003113

[3] Ikenaka T, Norioka S. Bowman-Birk family serine proteinase inhibitors. In: Barrett A. J.; Salvesen, editors. G (eds). ( (1986) Proteinase Inhibitors, Wagenigen, Elsevier Science cap. 9: 361–371.

[4] Laskowski M, Qasim MA (2000) What can the structures of enzyme-inhibitor complexes tell us about the structures of enzyme substrate complexes? Biochem Biophys Acta 1477: 324–337. 10708867 10.1016/s0167-4838(99)00284-8

[5] De Leo F, Volpicella M, Licciulli F, Liuni S, Gallerani R, Ceci LR (2002) PLANT-PIs: a database for plant protease inhibitors and their genes. Nucl Acids Res 30: 347–348. 11752333 10.1093/nar/30.1.347 PMC99076

[6] Ascenzi P, Bocedi A, Bolognesi M, Spallarossa A, Coletta M, et al (2003) The bovine basic pancreatic trypsin inhibitor (Kunitz inhibitor): a milestone protein. Curr Protein & Peptide Sci 4: 231–251. 12769721 10.2174/1389203033487180

[7] Joanitti GA, Freitas SM, Silva LP (2006) Proteinaceous Protease Inhibitors: Structural Features and Multiple Functional Faces. Current Enzyme Inhibition 2(3): 199–217.

[8] Mello MO, Tanaka AS, Silva-Filho MC (2003) Molecular evolution of Bowman-Birk type proteinase inhibitors in flowering plants. Mol Phylogenet Evol 27: 103–112. 12679075 10.1016/s1055-7903(02)00373-1

[9] Kennedy AR. (1993) Overview: anticarcinogenic activity of protease inhibitors. In: Troll W.; Kennedy, A. R. (eds). Protease inhibitors as Cancer Chemopreventive agents. New York: Plenum Press p. 9–64.

[10] Messina MJ, Persky V, Setchell KDR, Barnes S (1994) Soy intake and cancer risk: a review of the in vitro and in vivo data. Nutr Cancer 21: 113–131. 8058523 10.1080/01635589409514310

[11] Messina M, Barnes S (1991) The role of soy products in reducing risk of cancer. J Natl Cancer Inst 83: 541–546. 1672382 10.1093/jnci/83.8.541

[12] Kennedy AR (1998) The Bowman-Birk inhibitor from soybean as an anticarcinogenic agent. Am J Clin Nutr 68: 1406–1412. 10.1093/ajcn/68.6.1406S 9848508

[13] Kennedy AR (1995) Prevention of Carcinogenesis by Protease Inhibitors. Cancer Research (Suppl.) 54: 1999–2005. 8137328

[14] Joanitti GA, Azevedo RB, Freitas SM (2010) Apoptosis and lysosome membrane permeabilization induction on breast cancer cells by an anticarcinogenic Bowman–Birk protease inhibitor from *Vigna unguiculata* seeds. Cancer Letters. 293: 73–81. 10.1016/j.canlet.2009.12.017 20133052

[15] Clemente A, Sonnante G, Domoney C (2011) Bowman-Birk inhibitors from legumes and human gastrointestinal health: current status and perspectives. Curr Protein Pept Sci 12 (5): 358–373. 10.2174/138920311796391133 21418025

[16] Clemente A, Marín-Manzano MC, Jiménez E, Arques MC, Domoney C (2012) The anti-proliferative effect of TI1B, a major Bowman-Birk isoinhibitor from pea (Pisum sativum L.), on HT29 colon cancer cells is mediated through protease inhibition. Br J Nutr 108 Suppl. 1135–144. 10.1017/S000711451200075X 22916809

[17] Armstrong WB, Kennedy AR, Wan XS, Taylor TH, Nguyen QA, et al (2000) Clinical modulation of oral Leukoplakia and protease activity by Bowman–Birk inhibitor concentrate in a phase IIa chemoprevention trial. Clin Cancer Res 6: 4684–4691. 11156220

[18] Richardson M (1991) Seed storage proteins: the enzyme inhibitors. Meth Plant Biochem 5: 259–303.

[19] Bode W, Huber R (1992) Natural protein proteinase inhibitors and their interaction with proteinases. Eur J Biochem 204: 433–451. 1541261 10.1111/j.1432-1033.1992.tb16654.x

[20] Chen P, Rose J, Love R, Wei CH, Wang BC (1992) Reactive Sites of an Anticarcinogenic Bowman-Birk Proteinase Inhibitor are Similar to Other Trypsin Inhibitors. The Journal of Biological Chemistry 267: 1990–1994. 1730730 10.2210/pdb1pi2/pdb

[21] Clemente A, Marín-Manzano MC, Arques MC, Domoney C. (2013) Bowman-Birk Inhibitors from Legumes: Utilization in Disease Prevention and Therapy, Bioactive Food Peptides in Health and Disease. Dr. Blanca Hernández-Ledesma (Ed.), ISBN: 978–953–51–0964–8, In Tech, DOI: 10.5772/51262. http://www.intechopen.com/books/bioactive-food-peptides-in-health-and-disease/bowman-birk-inhibitors-from-legumes-utilisation-in-disease-prevention-and-therapy.

[22] Morhy L, Ventura MM (1987) The complete amino acid sequence of the *Vigna unguiculata* (L.) WaLP seed trypsin and chymotrypsin inhibitor. An Acad Bras Cienc 59: 71–81. 3426000

[23] Freitas SM, De Mello LV, Da Silva MC, Vriend G, Neshich G, et al (1997) Analysis of the black-eyed pea trypsin and chymotrypsin inhibitor-α-chymotrypsin complex. FEBS Lett 409: 121–127. 9202130 10.1016/s0014-5793(97)00419-5

[24] Barbosa JARG, Silva LP, Teles RCL, Esteves GF, Azevedo RB, et al (2007) Crystal Structure of the Bowman-Birk Inhibitor from *Vigna unguiculata* Seeds in Complex with b-Trypsin at 1.55 Å Resolution and Its Structural Properties in Association with Proteinases. Biophysical Journal 92: 1638–1650. 17142290 10.1529/biophysj.106.090555 PMC1796824

[25] Ventura MM, Martin CO, Morhy L (1975) A trypsin and chymotrypsin inhibitor from black-eyed pea (*Vigna sinensis* L.). VI. Isolation and properties of complexes with trypsin and chymotrypsin. An Acad Brasil Cienc 47: 335–346.

[26] Xavier-Filho J, Ventura MM (1988) Trypsin inhibitors in Cowpea: a review. Comments Agric Food Chem 1: 239–314.

[27] Fachetti HCS, Mizuta K, Ventura MM (1984) Thermodynamics of the black-eyed pea trypsin and chymotrypsin inhibitor. An Acad Bras Cienc 56: 311–317.

[28] Freitas SM, Ikemoto H, Ventura MM (1999) Thermodynamics of the binding of chymotrypsin with the black-eyed pea trypsin and chymotrypsin inhibitor (BTCI). J Protein Chem 18: 307–313. 10395449 10.1023/a:1021039429014

[29] Ventura MM, Xavier-Filho J (1966) A trypsin and chymotrypsin inhibitor from black-eyed pea (*V. Sinensis* L.) I. Purification and partial characterization. An Acad Bras Ciênc 38: 553–566.

[30] Silva LP, Leite JRSA, Bloch C, Freitas SM (2001) Stability of a Black Eyed Pea Trypsin/Chymotrypsin inhibitor (BTCI). Protein and Peptide Lett 8: 33–38.

[31] Franco OL, Santos RC, Batista JAN, Mendes ACM, Araújo A, et al (2003) Effects of black-eyed pea trypsin/chymotrypsin inhibitor on proteolytic activity and on development of *Anthonomus grandis* . Phytochemistry 63: 343–349. 12737983 10.1016/s0031-9422(03)00108-0

[32] Carvalho AF, Santos-Neto MS, Monteiro HSA, Freitas SM, Morhy L, et al (2008) BTCI enhances guanylin-induced natriuresis and promotes renal glomerular and tubular effects. Braz J Biol 68 (1): 149–154. 10.1590/s1519-69842008000100021 18470390

[33] Silva LP, Azevedo RB, Morais PC, Ventura MM, Freitas SM (2005) Oligomerization States of Bowman-Birk Inhibitor by Atomic Force Microscopy and Computational Approaches. Proteins: Structure, Function, and Bioinformatics 61: 642–648. 10.1002/prot.20646 16161117

[34] Esteves GF, Teles RCL, Calvacanti NS, Neves D, Ventura MM, et al (2007) Crystallization, data collection and processing of the chymotrypsin–BTCI–trypsin ternary complex. Acta Cryst F63: 1087–1090. 10.1107/S1744309107056424 PMC2344091 18084102

[35] Joanitti GA. Supervisors: Azevedo RB and Freitas SM. (2012) Ph thesis: Efeitos de extrato bruto de sementes de Vigna unguiculata e do inibidor de proteases BTCI, encapsulado em nanopartículas, no tratamento preventivo e terapêutico de câncer de mama e de pele, in vitro e in vivo. UnB-Depto de Morfologia.

[36] Hortobagyi GN, De La Garza Salazar J, Pritchard K, Amadori D, Haidinger R, et al (2005) The global breast cancer burden: variations in epidemiology and survival. Clin Breast Cancer 6: 391–401. 16381622 10.3816/cbc.2005.n.043

[37] Dou QP, Li B (1999) Proteasome inhibitors as potential novel anticancer agents. Drug Resist Updat 2 (4): 215–223. 10.1054/drup.1999.0095 11504494

[38] Bochtler M, Ditzel L, M, Hartmann C, Huber R (1999) The proteasome. Annu Rev Biophys Biomol Struct 28: 295–317. 10410804 10.1146/annurev.biophys.28.1.295

[39] Loidl G, Groll M, Musiol HJ, Huber R, Moroder L (1999) Bivalency as a principle for proteasome inhibition. Proc Natl Acad Sci USA 96 (10): 5418–5422. 10.1073/pnas.96.10.5418 PMC21874 10318898

[40] Glickman MH, Rubin DM, Fried VA, Finley D (1998) The regulatory particle of the *Saccharomyces cerevisiae* proteasome. Mol Cell Biol 18: 3149–3162. 9584156 10.1128/mcb.18.6.3149 PMC108897

[41] Voges D, Zwickl P, Baumeister W (1999) The 26S proteasome: a molecular machine designed for controlled proteolysis. Annu Rev Biochem 68: 1015–10168. 10872471 10.1146/annurev.biochem.68.1.1015

[42] Pickart CM, Cohen RE (2004) Proteasomes and their kin: proteases in the machine age. Nat Rev Mol Cell Biol 5: 177–187. 14990998 10.1038/nrm1336

[43] Hölzl H, Kapelari B, Kellermann J, Seemüller E, Sümegi M, et al (2000) The regulatory complex of Drosophila melanogaster 26S proteasomes. Subunit composition and localization of a deubiquitylating enzyme. J Cell Biol 150 (1): 119–130. 10.1083/jcb.150.1.119 PMC2185576 10893261

[44] Bossola M, Muscaritoli M, Costelli P, Grieco G, Bonelli G, et al (2003) Increased muscle proteasome activity correlates with disease severity in gastric cancer patients. Ann Surg 237: 384–389. 12616123 10.1097/01.SLA.0000055225.96357.71 PMC1514311

[45] Groll M, Ditzel L, Lowe J, Stock D, Bochtler M, et al (1997) Structure of 20S proteasome from yeast at 2.4 A° resolution. Nature 386: 463–471. 9087403 10.1038/386463a0

[46] Kloetzel PM, Ossendorp F (2004) Proteasome and peptidase function in MHC-class-I-mediated antigen presentation. Curr Opin Immunol 16: 76–81. 14734113 10.1016/j.coi.2003.11.004

[47] Blackburn C, Gigstad KM, Hales P, Garcia K, Jones M, et al (2010) Characterization of a new series of non-covalent proteasome inhibitors with exquisite potency and selectivity for the 20S β5-subunit.Biochem. J 430: 461–476. 10.1042/BJ20100383 PMC2933030 20632995

[48] Gavilán E, Sánchez-Aguayo I, Daza P, Ruano D (2013) GSK-3b signaling determines autophagy activation in the breast tumor cell line MCF-7 and inclusion formation in the non-tumor cell line MCF-10A in response to proteasome inhibition. Cell Death and Disease 4: e572. 23559006 10.1038/cddis.2013.95 PMC3668630

[49] Wehenkel M, Ban JO, Ho YK, Carmony KC, Hong JT, et al (2012) A selective inhibitor of the immunoproteasome subunit LMP2 induces apoptosis in PC-3 cells and suppresses tumour growth in nude mice. Br J Cancer 107: 53–62. 22677907 10.1038/bjc.2012.243 PMC3389428

[50] Park JE, Ao L, Miller Z, Kim K, Wu Y, et al (2013) PSMB9 Codon 60 Polymorphisms Have No Impact on the Activity of the Immunoproteasome Catalytic Subunit B1i Expressed in Multiple Types of Solid Cancer. PLoS One 8(9): e73732. 24040045 10.1371/journal.pone.0073732 PMC3767749

[51] Codony-Servat J, Tapia MA, Bosch M, Oliva C, Domingo-Domenech J, et al (2006) Differential cellular and molecular effects of bortezomib, a proteasome inhibitor, in human breast cancer cells. Mol Cancer Ther 5 (3): 665–675. 10.1158/1535-7163.MCT-05-0147 16546981

[52] An B, Goldfarb RH, Siman R, Dou QP (1998) Novel dipeptidyl proteasome inhibitors overcome Bcl-2 protective function and selectively accumulate the cyclin-dependent kinase inhibitor p27 and induce apoptosis in transformed, but not normal, human fibroblasts. Cell Death Differ 5: 1062–1075. 9894613 10.1038/sj.cdd.4400436

[53] Lopes UG, Erhardt P, Yao R, Cooper GM (1997) p53-dependent induction of apoptosis by proteasome inhibitors. J Biol Chem 272 (20): 12893–12896. 10.1074/jbc.272.20.12893 9148891

[54] Voorhees PM, Dees EC, O’Neil B, Orlowski RZ (2003) The Proteasome as a Target for Cancer Therapy. Clinical Cancer Research 9: 6316–6325. 14695130

[55] Chen YW, Huang S, Lin-Shiau S, LIN J (2005) Bowman–Birk inhibitor abates proteasome function and suppresses the proliferation of MCF-7 breast cancer cells through accumulation of MAP kinase phosphatase-1. Carcinogenesis 26 (7): 1296–1306. 10.1093/carcin/bgi062 15746161

[56] Demasi M, Shringarpure R, Davies KJ (2001) Glutathiolation of the proteasome is enhanced by proteolytic inhibitors. Arch Biochem Biophys 389 (2): 254–263. 10.1006/abbi.2001.2332 11339815

[57] Frisken BJ (2001) Revisiting the method of cumulants for the analysis of dynamic light-scattering data. Applied Optics 40 (24): 4087–4091. 10.1364/ao.40.004087 18360445

[58] Hassan PA, Kulshreshtha SK (2006) Modification to the cumulant analysis of polydispersity in quasielastic light scattering data. Journal of Colloid and Interface Science 300(2): 744–748. 16790246 10.1016/j.jcis.2006.04.013

[59] Baldwin ET, Crumley KV, Carter CW (1986) Practical, Rapid Screening of Protein Crystallization Conditions by Dynamic Light Scattering. Biophys J 49 (1): 47–48. 10.1016/s0006-3495(86)83587-1 PMC1329571 19431643

[60] Tardioli S, Bonincontro A, Mesa CL, Muzzalupo R (2010) Interaction of bovine serum albumin with gemini surfactants. Journal of Colloid and Interface Science 347 (1): 96–101. 10.1016/j.jcis.2010.03.017 20362296

[61] Boehm K, Guddorf J, Albers A, Kamiyama T, Fetzner S, Hinz HJ (2008) Thermodynamic Analysis of Denaturant-Induced Unfolding of HodC69S Protein Supports a Three-State Mechanism. Biochemistry 47: 7116–7126. 18549245 10.1021/bi800554v

[62] Carrotta R, Manno M, Giordano FM, Longo A, Portale G, et al (2009) Protein stability modulated by a conformational effector: effects of trifluoroethanol on bovine serum albumin. Phys Chem. Chem Phys 11: 4007–4018. 10.1039/b818687a 19440630

[63] Hanlon AD, Larkin MI, Reddick RM (2010) Free-Solution, Label-Free Protein-Protein Interactions Characterized by Dynamic Light Scattering. Biophysical Journal 98: 297–304. 20338851 10.1016/j.bpj.2009.09.061 PMC2808485

[64] Lacerda SH, Park JJ, Meuse C, Pristinski D, Becker ML, et al (2010) Interaction of gold nanoparticles with common human blood proteins. ACS Nano 4 (1): 365–379. 10.1021/nn9011187 20020753

[65] Liang L, Yao P, Gong J, Jiang M (2004) Interaction of apo cytochrome c with sulfonated polystyrene nanoparticles. Langmuir 20 (8): 3333–3338. 15875866

[66] Chen L, Tianqing L (2008) Interaction behaviors between chitosan and hemoglobin. Int J Biol Macromol 42 (5): 441–446. 10.1016/j.ijbiomac.2008.02.005 18394697

[67] Libich DS, Hill CM, Bates IR, Hallett FR, Armstrong S, et al (2003) Interaction of the 18.5-kD isoform of myelin basic protein with Ca2+-calmodulin: effects of deimination assessed by intrinsic Trp fluorescence spectroscopy, dynamic light scattering, and circular dichroism. Protein Sci 12 (7): 1507–1521. 10.1110/ps.0303603 PMC2323942 12824496

[68] Gokarn YR, Fesinmeyer RM, Saluja A, CAO S, Dankberg J, et al (2009) Ion-specific modulation of protein interactions: Anion-induced, reversible oligomerization of a fusion protein. Protein Science 18: 169–179. 19177361 10.1002/pro.20 PMC2708029

[69] Shiba K, Niidome T, Katoh E, Xiang H, Han L, et al (2010) Polydispersity as a Parameter for Indicating the Thermal Stability of Proteins by Dynamic Light Scattering. Analytical Sciences 26: 659. 20543496 10.2116/analsci.26.659

[70] Li S, Xing D, LI J (2004) Dynamic Light Scattering Application to Study Protein Interactions in Electrolyte Solutions. Journal of Biological Physics 30: 313–324. 23345875 10.1007/s10867-004-0997-z PMC3456317

[71] Kopp F, Steiner R, Dahlmann B, Kuehn L, Reinauer H (1986) Size and shape of the multicatalytic proteinase from rat skeletal muscle. Biochim Biophys Acta 872 (3): 253–260. 10.1016/0167-4838(86)90278-5 3524688

[72] Yoshimura T, Kameyama K, Takagi T, Ikai A, Tokunaga F, et al (1993) Molecular characterization of the “26S” proteasome complex from rat liver. J Struct Biol 111 (3): 200–211. 10.1006/jsbi.1993.1050 8003381

[73] Ventura MM, Mizuta K, Ikemoto H (1981) Self-association of the black-eyed pea trypsin and chymotrypsin inhibitor in solution - a study by light-scattering. An Acad Bras Cienc 53: 195–201.

[74] Lupas A, Koster AJ, Baumeister W (1993) Structural features of 26S e 20S proteasomes. Enzyme Protein 47: 252–273. 7697124 10.1159/000468684

[75] Tanaka K, Yoshimura T, Kumatori A, Ichihara A, Ikai A, et al (1988) Proteasomes (multi-protease complexes) as 20 S ring-shaped particles in a variety of eukaryotic cells. J Biol Chem 263 (31): 16209–16217. 3141402

[76] Thomas AR, Oosthuizen V, Naudé RJ, Muramoto K (2002) Purification and characterization of the 20S proteasome from ostrich skeletal muscle. Biol Chem 383 (7–8): 1267–1270. 10.1515/BC.2002.141 12437115

[77] Griffin TA, Nandi D, Cruz M, Fehling HJ, Kaer LV, et al (1998) Immunoproteasome assembly: cooperative incorporation of interferon gamma (IFN-gamma)-inducible subunits. J Exp Med 187: 97–104. 9419215 10.1084/jem.187.1.97 PMC2199179

[78] Orlowski M, Wilk S (2000) Catalytic Activities of the 20S proteasome, a multicatalytic proteinase complex. Arch Biochem Biophys 383 (1): 1–16. 10.1006/abbi.2000.2036 11097171

[79] Groll M, Huber R (2003) Substrate access and processing by the 20S proteasome core particle. Internat J Biochem 35: 606–616. 10.1016/s1357-2725(02)00390-4 12672453

[80] McBride JD, Leatherbarrow RJ (2001) Synthetic Peptide Mimics of the Bowman-Birk Inhibitor Protein Current Medicinal Chemistry. 8: 909–917. 10.2174/0929867013372832 11375759

[81] deBettignies G, Coux O (2010) Proteasome inhibitors: dozens of molecules and still counting. Biochimie 92: 1530–45. 20615448 10.1016/j.biochi.2010.06.023

[82] Kisselev AF, van der Linden WA, Overkleeft HS (2012) Proteasome inhibitors: an expanding army attacking a unique target. Chem Biol 19: 99–115. 22284358 10.1016/j.chembiol.2012.01.003 PMC3503453

[83] Borissenko L, Groll M (2007) 20S proteasome and its inhibitors: crystallographic knowledge for drug development. Chem Rev 107: 687–717. 17316053 10.1021/cr0502504

[84] Kisselev AF, Kaganovich D, Goldberg AL (2002) Binding of Hydrophobic Peptides to Several Non-catalytic Sites Promotes Peptide Hydrolysis by All Active Sites of 20S Proteasomes. J Biol Chem 277: 22260–22270. 11927581 10.1074/jbc.M112360200

[85] Rock KL, Gramm C, Rothstein L, Clark K, Stein R, et al (1994) Inhibitors of the proteasome block the degradation of most cell proteins and the generation of peptides presented on MHC class I molecules. Cell 78: 761–771. 8087844 10.1016/s0092-8674(94)90462-6

[86] Pereira MEF, Chen WE, Li J, Johdo O (1996) The Antitumor Drug Aclacinomycin A, Which Inhibits the Degradation of Ubiquitinated Proteins, Shows Selectivity for the Chymotrypsin-like Activity of the Bovine Pituitary 20 S Proteasome. J Biol Chem 271: 16455–16459. 8663210 10.1074/jbc.271.28.16455

[87] Lee DH, Goldberg AL (1998) Proteasome inhibitors: valuable new tools for cell biologists. Trends in Cell Biology 8: 397–403. 9789328 10.1016/s0962-8924(98)01346-4

[88] Craiu A, Gaczynska M, Akopian T, Gramm CF, Fenteany G, et al (1997) Lactacystin and clasto-lactacystin beta-lactone modify multiple proteasome beta-subunits and inhibit intracellular protein degradation and major histocompatibility complex class I antigen presentation. J Biol Chem 272 (20): 13437–13445. 10.1074/jbc.272.20.13437 9148969

[89] Heinemeyer W, Fischer M, Krimmer T, Stachon U, Wolf DH (1997) The active sites of the eukaryotic 20 S proteasome and their involvement in subunit precursor processing. J Biol Chem 272: 25200–25209. 9312134 10.1074/jbc.272.40.25200

[90] Kisselev AF, Callard A, Goldberg AL (2006) Importance of the different proteolytic sites of the proteasome and the efficacy of inhibitors varies with the protein substrate. J Biol Chem 281 (13): 8582–8590. 10.1074/jbc.M509043200 16455650

[91] Schmidtke G, Kraft R, Kostka S, Henklein P, Frömmel C, et al (1996) Analysis of mammalian 20S proteasome biogenesis: the maturation of beta-subunits is an ordered two-step mechanism involving autocatalysis. The EMBO J 15: 6887–6898. 9003765 PMC452515

[92] Groll M, Heinemeyer W, Jäger S, Ullrich T, Bochtler M, et al (1999) The catalytic sites of 20S proteasomes and their role in subunit maturation: A mutational and crystallographic study, Proc Natl Acad Sci USA. 96: 10976–10983. 10.1073/pnas.96.20.10976 PMC34229 10500111

[93] Ghobrial IM, Witzig TE, Adjei AA (2005) Targeting apoptosis pathways in cancer therapy. CA Cancer J Clin 55 (3): 178–194. 10.3322/canjclin.55.3.178 15890640

[94] Shen M, Schmitt S, Buac D, Ping Dou Q (2013) Targeting the ubiquitin–proteasome system for cancer therapy. Expert Opin Ther Targets 17(9): 1091–1108. 23822887 10.1517/14728222.2013.815728 PMC3773690

[95] Mortenson MM, Schlieman MG, Virudachalam S, Bold RJ (2004) Effects of the proteasome inhibitor Bortezomib alone and in combination with chemotherapy in the A549 non-small-cell lung cancer cell line. Cancer Chemo Ther Pharmacol 54 (4): 343–353. 10.1007/s00280-004-0811-4 15197486

[96] Adams J, Palombella VJ, Sausville EA, Johnson J, Destree A, et al (1999) Proteasome inhibitors: a novel class of potent and effective antitumor agents. Cancer Res 59: 2615–2622. 10363983

[97] Adams J (2004) The proteasome: a suitable antineoplastic target. Nat Rev Cancer 4: 349–360. 15122206 10.1038/nrc1361

[98] Crawford LJ, Walker B, Ovaa H, Chauhan D, Anderson KC, et al (2006) Comparative selectivity and specificity of the proteasome inhibitors BzLLLCOCHO, PS-341, and MG-132. Cancer Res 66: 6379–6386. 16778216 10.1158/0008-5472.CAN-06-0605

[99] O’Connor OA, Wright J, Moskowitz C, Muzzy J, MacGregor-Cortelli B, et al (2005) Phase II clinical experience with the novel proteasome inhibitor bortezomib in patients with indolentnon-Hodgkin’s lymphoma and mantle cell lymphoma. J Clin Oncol 23: 676–684. 15613699 10.1200/JCO.2005.02.050

